# Peripheral Afferent Mechanisms Underlying Acupuncture Inhibition of Cocaine Behavioral Effects in Rats

**DOI:** 10.1371/journal.pone.0081018

**Published:** 2013-11-18

**Authors:** Seol Ah Kim, Bong Hyo Lee, Jong Han Bae, Kwang Joong Kim, Scott C. Steffensen, Yeon-Hee Ryu, Joong Woo Leem, Chae Ha Yang, Hee Young Kim

**Affiliations:** 1 College of Korean Medicine, Daegu Haany University, Daegu, South Korea; 2 Department of Physics, Yeungnam University, Gyeongsan, Gyeongbuk, South Korea; 3 Department of Psychology and Neuroscience, Brigham Young University, Provo, Utah, United States of America; 4 Acupuncture, Moxibustion & Meridian Research Center, Division of Standard Research, Korea Institute of Oriental Medicine, Daejeon, South Korea; 5 Department of Physiology, Yonsei University College of Medicine, Seoul, South Korea; Charité University Medicine Berlin, Germany

## Abstract

Administration of cocaine increases locomotor activity by enhancing dopamine transmission. To explore the peripheral mechanisms underlying acupuncture treatment for drug addiction, we developed a novel mechanical acupuncture instrument (MAI) for objective mechanical stimulation. The aim of this study was to evaluate whether acupuncture inhibition of cocaine-induced locomotor activity is mediated through specific peripheral nerves, the afferents from superficial or deep tissues, or specific groups of nerve fibers. Mechanical stimulation of acupuncture point HT7 with MAI suppressed cocaine-induced locomotor activity in a stimulus time-dependent manner, which was blocked by severing the ulnar nerve or by local anesthesia. Suppression of cocaine-induced locomotor activity was elicited after HT7 stimulation at frequencies of either 50 (for Meissner corpuscles) or 200 (for Pacinian corpuscles) Hz and was not affected by block of C/Aδ-fibers in the ulnar nerve with resiniferatoxin, nor generated by direct stimulation of C/Aδ-fiber afferents with capsaicin. These findings suggest that HT7 inhibition of cocaine-induced locomotor activity is mediated by A-fiber activation of ulnar nerve that originates in superficial and deep tissue.

## Introduction

Acupuncture is widely practiced for treating many functional disorders such as substance abuse, pain and psychological problems, although there are still many unresolved issues related to its effectiveness. In 1997, the NIH released a consensus statement concluding that acupuncture is effective or at least useful for the treatment of 13 conditions including drug addiction, low back pain and stroke rehabilitation [1]. Our previous studies have shown that manual acupuncture at acupoint *Shenmen* (HT7), located at the ulnar side of the wrist, suppresses drug self-administration behavior or relapse to abused drugs such as cocaine, morphine and ethanol [2-5]. The central mechanism underlying acupuncture’s role in suppressing the reinforcing effects of abused drugs includes modulation of GABAergic neurons in the ventral tegmental area (VTA) through opioid receptors [3,4] and suppression of dopamine (DA) release in the nucleus accumbens (NAc) [2,4]. We have proposed that acupuncture stimulates opioidergic neurons in the arcuate nucleus of hypothalamus and that opioids released from the arcuate nucleus can activate opioid receptors on accumbal GABA terminals leading to a net decrease of DA transmission and the reinforcing effects of abused drugs [2,5,6]. Although the results from animal and human studies provide evidence that HT7 acupuncture produces reliable and reproducible effects on various addictive behaviors [3,7-9], these studies have focused primarily on the central mechanism underlying HT7 acupuncture. The peripheral mechanism of acupuncture in addiction remains to be elucidated. Previous studies have shown that specific types of afferent fibers mediate acupuncture’s effects. For example, acupuncture analgesia following electrical stimulation to ST36 requires activation of A-fibers [10], and acupuncture applied to PC5-6 acupoints activates Aδ and C fibers to evoke cardiovascular effects [11]. The present study investigated the peripheral mechanism underlying the effectiveness of manual acupuncture on cocaine- induced locomotion. To overcome the variability in depth, intensity and/or duration of stimulation typically inherent in manual acupuncture studies, we developed a novel device (mechanical acupuncture instrument; MAI) which was able to apply a predictable and reproducible stimulation to acupuncture sites. To evaluate its effectiveness, we hypothesized that MAI HT7 acupuncture would: 1) Suppress cocaine-induced locomotor activity; 2) Be mediated by specific fiber types in the ulnar nerve; 3) Be unaffected by C/Aδ-fiber activation; and 4) Be mediated by fast fiber types in the ulnar nerve.

## Materials and Methods

### Animals and Ethics Statement

Male Sprague-Dawley rats (weight 270-320g, Daehan Animal, Seoul, Korea) were used. All rats had free access to food and water and maintained on a 12 hr light-dark cycle. All procedures were approved by the Institutional Animal Care and Use Committee at Daegu Haany University (DHU2012-008). Each group consisted of 6–8 rats, unless stated otherwise. 

### Chemicals

Resiniferatoxin (RTX; Sigma-Aldrich, MO, USA), an ultrapotent capsaicin analogue known to block afferents containing transient receptor potential vanilloid receptor (mostly C- and Aδ-fibers) [12], was dissolved at 0.01 % concentration in vehicle solution that contained 0.3% Tween 80, 10% DMSO and saline. 1,19-dioleyl-3,3,39,3-tetramethy lindocarbocyanine methanesulfonate (DiI; 25mg, Life Technologies, USA), a retrograde tracer, was dissolved in 0.5 ml methanol. Bupivacaine (0.5 %, Huons Pharm, Korea), a long-acting local anesthetic and cocaine hydrochloride (Macfarlan Smith Ltd., Edinburgh, UK) were dissolved in saline. Capsaicin (0.05 %, 10 ul; Sigma-Aldrich, MO, USA) was dissolved in a vehicle consisting of 10 % alcohol and 10 % Tween 80 in 0.9 % saline. 

### Mechanical Acupuncture Instrument (MAI) and Acupuncture Treatment

**Figure 1:**
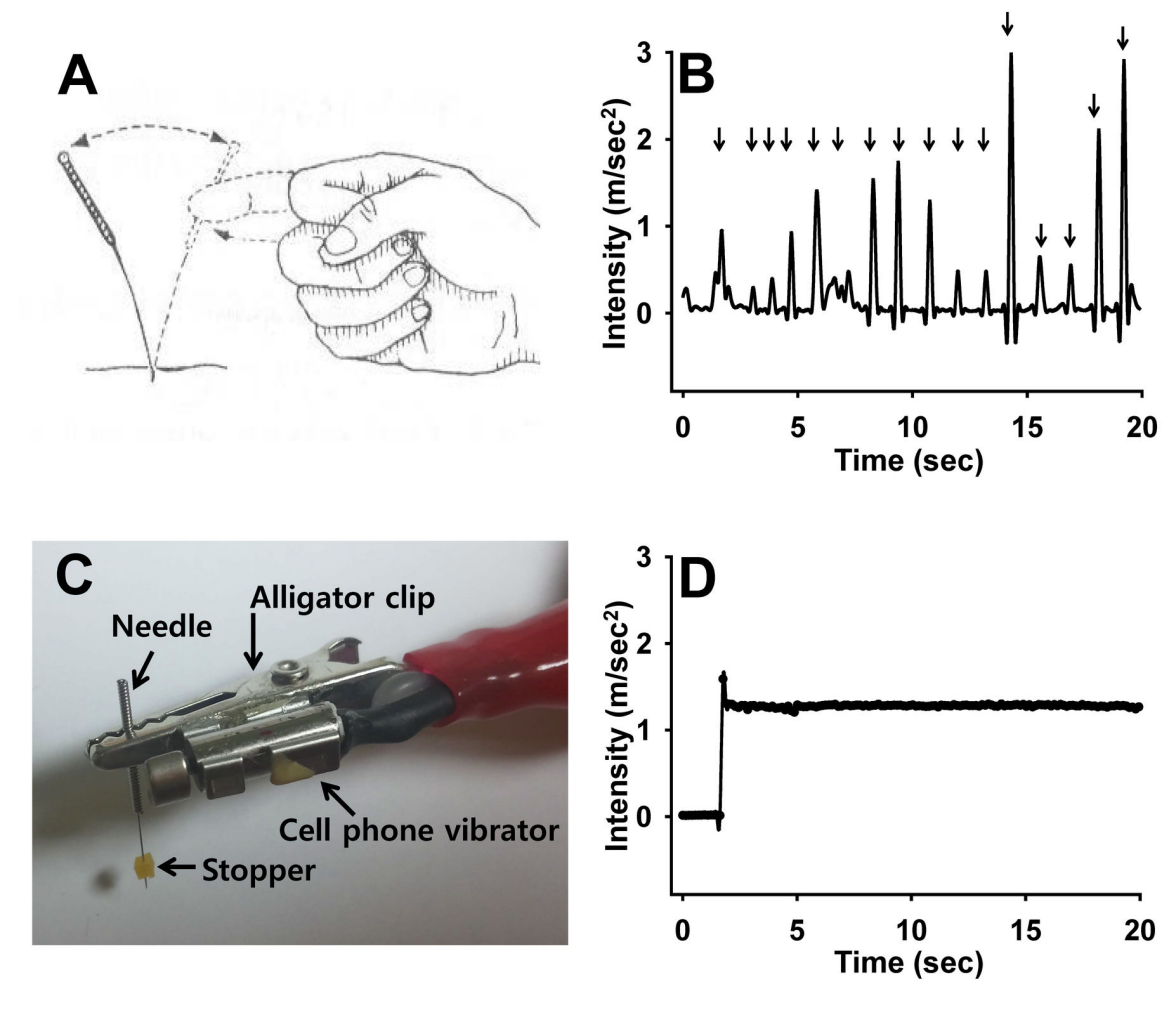
Mechanical acupuncture instrument (MAI). **A**, **B**: In traditional manual acupuncture, a needle is manually stimulated by manipulating the handle of the needle with an index finger after being inserted into acupoints (A). Manipulating a needle (arrows) elicited abrupt increases of intensity, but with considerable variations in peak values of intensity (B). **C**, **D**: With the novel MAI, a cell phone motor is connected to the acuneedle to create a constant vibration of the needle and operated by a control unit for adjustment of intensity (acceleration), frequency and operation time (C). The acceleration parameter of 1.3 m/sec^2^ in intensity and 85 Hz in frequency was routinely used throughout our experiments (D).

A novel mechanical acupuncture instrument (MAI) was developed to mimic the vibrations produced by manual acupuncture stimulation. This device consisted of a custom-made control unit and a cell phone vibrator (MB-0412V or MB-1203V, Motor bank, Korea) mated to an acupuncture needle (Figure 1C). To control the depth of acupuncture needle insertion (0.10 mm in diameter, 10 mm in length of needle and 10 mm in length of handle; Dongbang Medical Co., Korea), a rubber grommet was fixed to the needle at a distance of 1 or 3 mm from the tip. The needles were inserted into acupoints, vibrated with MAI for 10-40 seconds, maintained up to 1 min after needle insertion and subsequently withdrawn. Positioning of acupoints at HT7 or LI5 was based on the transpositional method, which locates animal acupoints on the surface of their skin corresponding to the anatomic site of human acupoints [13]. HT7 was identified at the transverse crease of the wrist of the forepaw, radial to the tendon of the flexor carpi ulnaris muscle. To control the possibility of locomotor disturbance by motor impairment in the wrist by acupuncture at HT7, a nearby point at wrist LI5 was chosen as the corresponding control point to HT7 at the opposite side of the wrist, about 5 mm apart from HT7, located at the distal end of the radius between the tendons of the palmaris longus muscle and flexor carpi radialis muscle proximal to the transverse crease of the wrist of the forepaw. One day prior to testing, all rats were habituated to the experimental procedures which included handling, acupuncture manipulation without needle insertion (2-3 min) and exposure to locomotor cages (90 min). One minute after cocaine injection, acupuncture was applied bilaterally at acupoints on wrists for 1 min while an assistant lightly restrained the rat. The animals in non-acupuncture groups were lightly restrained in the same manner as the acupuncture treatment, but without needle insertion.

### Measurement of Intensity and Frequency of Mechanical Stimulation

To measure intensity and frequency of mechanical stimulation (vibration) by MAI, a tip acupuncture needle was attached to an accelerometer (PO-AXA-12-01, Intellane co., Korea) which converted acceleration or frequency from motion to voltage. The signals from the accelerometer were digitized using a data acquisition card (DAQ-NI USB-6200, National Instruments, Austin, TX, USA) and sent to a customized LabVIEW (National Instruments, Austin, TX, USA) virtual instrument software program for analysis of intensity (acceleration) and frequency of vibration. 

### Cocaine-induced Locomotor Activity

Locomotor activity was measured by an image analysis system (Ethovision, Noldus Information Technology BV, Wageningen, Netherlands). In a dimly-lit room, each animal was placed in a square open field box (40 cm × 40 cm× 45cm) made of black acrylic. Animal activity was monitored with a video camera mounted above the box. Video tracking software (Ethovision 3.1) measured the distance travelled (cm) by the animal. On the testing day, animals were habituated for 60 min in the open field. After recording baseline activity for 30 min, the animal was given an intraperitoneal injection of cocaine (15 mg/kg) and monitored for up to 60 min after injection. The distance travelled during each 10-min period was analyzed. Data were expressed as a percentage of baseline or total travelled distance (cm) for 1 hour. 

### Surgical Transection of Radial, Median, or Ulnar Nerves

To identify the peripheral nerves mediating the acupuncture effects of HT7, major peripheral nerves innervating the forearm were selectively cut near the elbow, as described in detail [14,15]. Rats (n= 44) were randomized as 4 groups: (1) Sham (Con; n=12); (2) HT7 (HT7; n=12); (3) Ulnar nerve (Ulnar+HT7; n=11); and (4) Radial/Median nerves (Rad/Med+HT7; n=9) groups. For nerve surgical transection, a small skin incision was made under isoflurane anesthesia longitudinally on the medial part of elbow to expose radial, median and ulnar nerves. In the Radial/Median nerve group (Rad/Med+HT7), both the median and radial nerves were bilaterally ligated with 4-0 silk and cut distally. In the Ulnar nerve group (Ulnar+HT7), the ulnar nerve was cut around the medial head of the triceps muscle of both forelimbs. In the Sham group (Con), the nerves were exposed but not dissected. All incisions were closed following the surgical procedure. Forty eight to 72 hours after peripheral nerve injury, the rats were subjected to the locomotor activity test following acute cocaine injection. 

### Perineural Injection of Resiniferatoxin into Ulnar Nerve

To determine which afferent fibers, large A- or C/Aδ- fibers, mediate the acupuncture effects of HT7, a specific C/Aδ-fiber blocker resiniferatoxin (RTX) was administered perineurally in the ulnar nerve of both forelimbs, as described previously [16]. In brief, a stock solution (1 μg/μl) of RTX was prepared in 100 % dimethyl sulfoxide (DMSO; Sigma, USA), aliquoted and stored in a deep freezer. It was diluted to concentration of 0.01% in saline containing 0.3% Tween 80 prior to use. Under isoflurane anesthesia, after skin incision over the anterior part of the upper forelimb, the tensor fasciae antebrachii muscle was then separated with iris scissors to expose the ulnar nerve. RTX (0.01%, 100 μl) was then injected into the perineural space of ulnar nerve (RTX+HT7 group). The Control group (Con) received the same treatment as RTX-injected rats, except they received a perineural injection of vehicle (0.3% Tween 80, 10% DMSO, and 0.9% NaCl). Locomotor activity tests were performed within 48-72 hours after RTX or vehicle injection. 

Another set of experiments was carried out to confirm blockade of C-fiber transmission by RTX, by using an IB4 tracing method [17]. In brief, 48 hours after perineural injection of either RTX or vehicle into the right and left ulnar nerve, respectively, an FITC IB4 tracer (FITC-conjugated isolectin B4; 1%, 3 μl, Vector Laboratories, Burlingame, USA) was administered into ulnar nerve bilaterally under isoflurane anesthesia in rats (n=2). Three days later, C8 and T1 dorsal root ganglion (DRG) neurons of were excised, postfixed in 4 % buffered paraformaldehyde (PFA) for 2 hours, immersed in 30 % sucrose overnight and cryosectioned at 30 μm. The cryosections were then mounted on gelatin-coated glass slides. C8 and T1 DRGs labeled by IB4 were imaged under an Olympus AX70 fluorescence microscope (Olympus, Japan). 

### Afferent Neuronal Profiles Detected by Injection of DiI into HT7

To characterize the types of afferent fibers innervating HT7 acupoints, a retrograde tracer DiI was injected into the muscle [16] below the skin of HT7 acupoints and retrogradely labeled DRG (dorsal root ganglion) neurons were analyzed for cell size distribution and further identified with specific neurochemical marker for A-or C-fibers. Briefly, under isoflurane anesthesia, DiI (10 μl) was injected 3 mm deep below the skin of HT7 acupoints or adjacent muscle (control; midpoint of flexor carpi ulnaris), using a fine syringe with 27-gauge needle. Two weeks later, the rats were anesthetized with pentobarbital sodium (90 mg/kg, i.p.) and perfused to isolate dorsal root ganglion (DRG) neurons of C8 and T1 bilaterally. The DRGs were postfixed in 4 % buffered PFA for 2 hours, immersed in 30 % sucrose overnight and cryosectioned at 30 μm. The cryosections were then mounted on gelatin-coated glass slides. The C8 and T1 DRGs labeled by DiI were imaged under an Olympus AX70 fluorescence microscope (Olympus, Japan). The size of DiI-positive DRG neurons was measured using image analysis software (i-solution, IMT, Korea). To evaluate the fiber types innervating HT7 acupoints, the DiI-positive DRGs were double-stained using neurofilament 200 (NF200; Sigma, Saint Louis, USA), an immunohistochemical marker for A-fibers, or FITC-conjugated isolectin B4 (FITC IB4), a marker for C-fibers. Sections of C8 or T1 DRGs on gelatin-coated slides were incubated with NF200 (1:2000) or FITC IB4 (1:100). The sections incubated with NF200 were further processed with a secondary antibody, goat anti-mouse Alexa Fluor 488 (green; 1:200, Sigma-Aldrich, MO, USA). Each tissue section was imaged with a 20X objective using a confocal fluorescent imaging system (Leica TCSK-SP5-II) attached to a DM 6000-CFS upright microscope.

To test if the DiI-positive DRGs were activated by HT7 MAI stimulation, we assessed the expression of neuronal activation marker c-Fos in the DiI-positive DRGs in two rats. Two weeks after DiI injection into HT7, MAI stimulation for 60 sec was applied to left HT7 and the right HT7 was untreated to compare as control. Twenty min later, the animals were perfused and DRGs of C8 and T1 (left n=4, right n=4) were taken out for c-Fos immunohistochemistry. Sections of DRGs on gelatin-coated slides were incubated with c-Fos (1:1000; rabbit polyclonal antibody sc-52, Santa Cruz Biotechnology, Santa Cruz, USA) followed by Alexa Fluor 488 goat anti-rabbit (green; 1:200, Sigma-Aldrich, MO, USA). Each tissue section was imaged under an Olympus AX70 fluorescence microscope (Olympus, Japan). The number of c-Fos labeled neurons was compared between left (HT7-MAI stimulation) and right (control; no treatment) DRGs. Data for c-Fos are expressed as a percentage of c-Fos expressing cells per DiI positive neurons. 

### Somatic Afferent Recordings

To verify which types of fibers were activated by MAI stimulation of HT7, single-unit recordings were performed as described previously [11,18,19]. The right ulnar nerve was exposed between the medial belly of triceps muscle and humerus condyle and covered with mineral oil contained by skin flaps. The nerve was placed on a small mirror-based platform and after the perineural sheath was removed, the nerve was teased into fine bundles and split to further obtain a fine filament under surgical microscope. The filament was looped around a silver-wire recording electrode and the discharge activity was preamplified (gain 1000) and filtered at 5-1000 Hz by an ISO-80 amplifier (World Precision Instruments, USA). The signals were subsequently amplified (gain 10), notch filtered, digitized using a Cyberamp 320 signal conditioner (Axon Instruments Inc., USA) and CED1401plus (Cambridge Electronic Design, Cambridge, UK) and analyzed with software (Spike2 version 7, Cambridge Electronic Design, Cambridge, UK). To ensure the unit activity was from afferent fibers innervating HT7 acupoint, the action potential was evoked by mechanically stimulating HT7 using a camel-hair brush, von Frey filament (8 gram force) or blunt-tipped forceps. The conduction velocity of fibers was measured by dividing the conduction distances by the latency after the electrical stimulation artifact. Fiber types were classified according to conduction velocity as described in previous studies of rats [20]: >14 m/sec for Aβ, 2.2–14 m/sec for Aδ, and <1.4 m/sec for C fibers. Action potentials of 25 afferent fibers (14 Aβ, 1 Aδ and 10 C afferents) serving HT7 area from 5 rats were recorded before and during MAI stimulation of 10 sec at HT7. To further confirm the activation of peripheral receptor of A-fiber during HT7 MAI stimulation, the units of afferent fibers from Pacinian or Meissner corpuscles were identified as described in previous studies [18,19] and the effects of MAI HT7 on the unit activity were examined. Pacinian corpuscle units responded to high-frequency mechanical vibration with a tuning fork (256 Hz) and had a large receptive field (>6 mm) diffused in and around HT7, whereas Meissner corpuscle units had transient responses at the beginning and the end of the sustained pressure on HT7 for about 6-8 sec with von Frey 8 gram force.

### Data Analysis

Data are presented as mean ± SEM (standard error of the mean) and analyzed by one- or two-way repeated-measurement analysis of variance (ANOVA), followed by post hoc testing using the Holm-Sidak method or paired Student’s t-test, where appropriate. P≤ 0.05 was considered statistically significant. 

## Results

### Simple Acupuncture Device for Objective Mechanical Needle Stimulation

Manual manipulation following needle insertion has generally been used to produce acupuncture effects. However, the control of manual stimulation is subject to many vagaries including stimulation frequency and intensity, leading to low reproducibility and high individual variations among physicians [21]. To mimic mechanical stimulations generated by manual stimulation (Figure 1A), we developed a simple mechanical acupuncture instrument (MAI; Figure 1C). This device was designed to stimulate acupuncture needle mechanically with a cell phone vibrator (Figure 1C). The intensity and time of mechanical stimulation were regulated by a motor controller. First, to determine the strength and frequency of mechanical stimulations produced by manual acupuncture, an acupuncture needle (0.10 mm in diameter, 10 mm in length of needle shaft and 10 mm in length of handle part; Dongbang Medical Co., Korea) was fixed to one side of an accelerometer connected to a data acquisition system. Five volunteers including 2 well-trained acupuncturists and 3 persons with experience in animal acupuncture experiments were asked to manipulate the handle of the acupuncture needle with their index finger (Figure 1A). Intensity and frequency of mechanical stimulation transferred to the needle tip was recorded. As shown in Figure 1B, manual manipulation elicited a sharp increase in intensity (acceleration) followed by a rapid drop in intensity. Intensity (90 trials from 5 operators) ranged from 0.1 to 2.9 (mean value of 1.3 m/sec^2^). Vibration frequencies ranging from 70 ~ 90 Hz were detected in the tip of acupuncture needle upon manipulation (data not shown). However, considerable variations in intensity and frequency were observed within each operator or across operators. Based on these findings with operators, the mechanical acupuncture instrument was preset at 1.3 m/sec^2^ in intensity and 85 Hz in frequency for subsequent experiments (Figure 1D). 

### Acupuncture Suppressed Cocaine-induced Locomotor Activity in a Stimulus Time-dependent Manner

**Figure 2:**
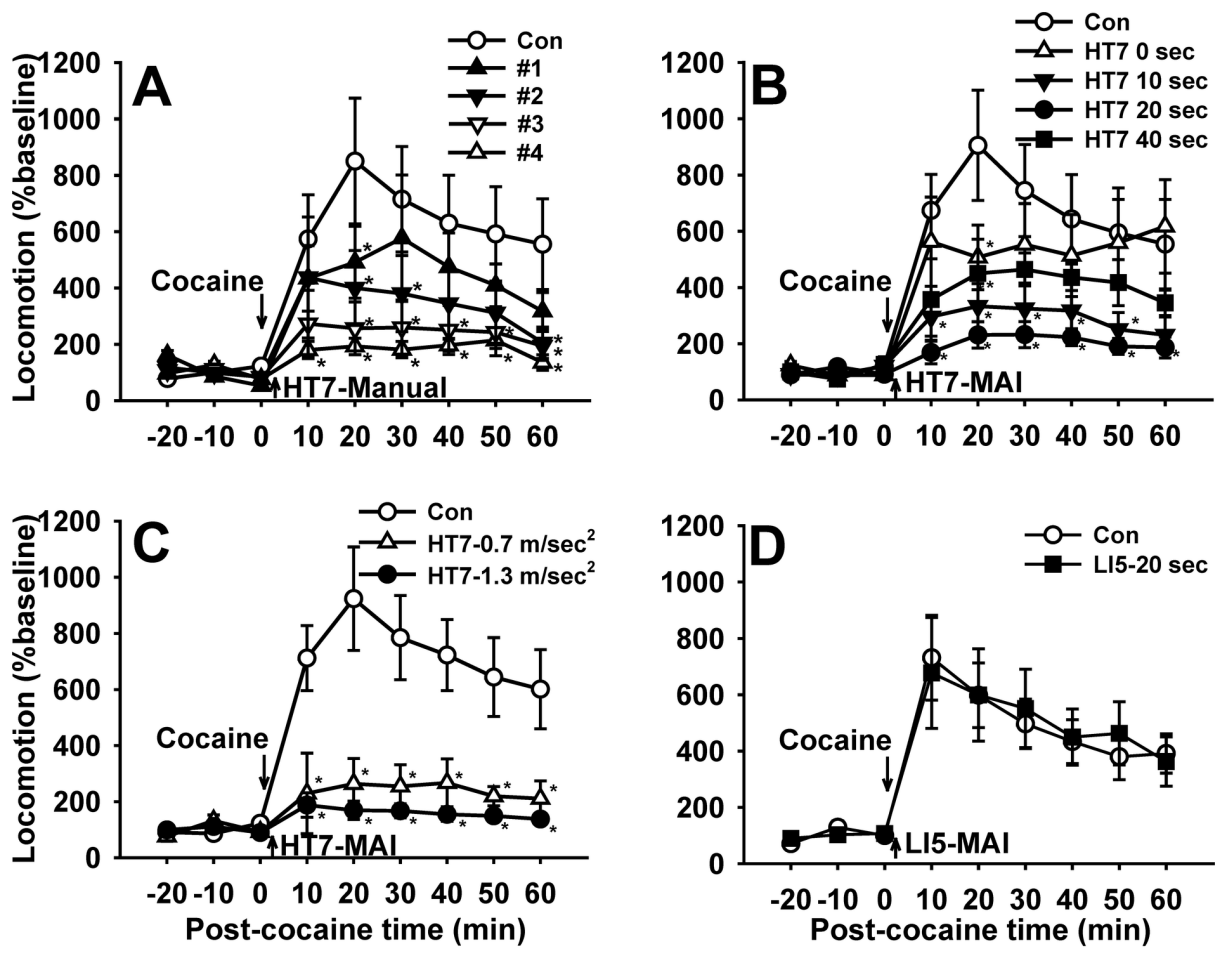
Mechanical acupuncture by MAI decreases cocaine-induced locomotor activity in a stimulus time-dependent manner. A: Effect of manual acupuncture at HT7 by 4 different operators (#1 to #4) on cocaine-induced locomotor activity. Acupuncture was performed by 4 operators consisting of 2 beginners (#1, #2) and 2 well-trained operators with experience over 2 years in acupuncture experiments (#3, #4). Variations in the effectiveness of acupuncture on cocaine locomotion are seen among experimenters. B, C: Effect of mechanical stimulation applied to HT7 with MAI on cocaine locomotion. Acupuncture needles were mechanically stimulated at different times (0, 10, 20 or 40 sec) with MAI (1.3 m/sec^2^ in intensity and 85 Hz in frequency). The acupuncture-induced inhibition of cocaine locomotion was stimulus-time dependent, reaching a maximum at 20-sec duration (B). Two different intensities (0.9 or 1.3 m/sec^2^) were applied for 20 sec to the needles inserted at a depth of 3 mm into HT7. The effect was produced with two different intensities of mechanical stimulation, either 0.7 (low) or 1.3 (high) m/sec^2^ at 20-sec duration, compared to control (cocaine only) (C). D: Effect of mechanical acupuncture at control point LI5 with MAI on cocaine locomotion. No acupuncture effect was produced when a parameter of 20-sec duration and 1.3 m/sec^2^ intensity was applied to LI5 acupoint at 85 Hz. n= 6-8 rats for each group. Data are presented as mean ± SEM and analyzed by two-way repeated ANOVA with Holm-Sidak post hoc comparison. *p<0.05 compared to control (cocaine only).

In our previous studies using manual acupuncture, one of the critical problems has been the inter-operator variations in the effectiveness of acupuncture. For this reason, acupuncture is routinely performed by well-trained operators or acupuncturists to minimize variability. We evaluated whether manual acupuncture could suppress acute cocaine-induced locomotor activity. Acupuncture was performed by 4 operators consisting of 2 beginners (#1, #2) and 2 well-trained operators with experience over 2 years in acupuncture experiments (#3, #4; Figure 2A). After baseline recording of locomotor activity (travelled distance) for 30 min, the rats were injected with cocaine (15 mg/kg). One min after injection, acupuncture needles were inserted at a depth of 3 mm into acupoints HT7, manually stimulated, as previously reported [3], and withdrawn 1 min after insertion of the needle. Locomotion increased rapidly after an acute injection of cocaine, which lasted up to about 60 min with a peak at 20 min (Con group in Figure 2A). Manual stimulation of needles inserted into HT7 inhibited the increase of locomotion produced by cocaine (time factor of two-way repeated measures ANOVA, F=3.501, p=0.010; Holm–Sidak post-hoc, *p<0.05 vs. Con). However, as expected, there were variationsbetween beginners (#1, #2) and well-trained operators (#3, #4) in the acupuncture effects, especially on time points of 20 (p=0.048) and 30 min (p=0.022) (two-way repeated measures ANOVA: group factor F=4.245, p=0.073; time factor F=3.501, p=0.010; interaction F=1.409, p=0.242; Figure 2A). To ameliorate operator variations in manual acupuncture treatments, we evaluated the utility of MAI to determine if the acupuncture effect was dependent on stimulus-time. After injecting cocaine, acupuncture needles were inserted into HT7 and mechanically stimulated at different times (0, 10, 20 or 40 sec) with MAI (1.3 m/sec^2^ in intensity and 85 Hz in frequency), maintained up to 1 min after insertion of the needles and then withdrawn. In 0-, 10- and 20-sec groups, the increase of cocaine locomotion was significantly inhibited in a stimulus time-dependent manner (two-way repeated measures ANOVA: group factor F=3.255, p=0.082; time factor F=11.611, p<0.001; interaction F=2.222, p=0.022; Holm–Sidak post-hoc, *p<0.05 vs. Con; Figure 2B). Two way ANOVA showed significant effects between stimulus time and treatment groups (P<0.05). Although the rats had been trained to tolerate human handling and restraint during acupuncture procedure, stressful behaviors such as avoidance and vocalization during continuous 40-sec vibration were seen (HT7-40 sec group), which might lead to lesser effects than those of other treatment times. 

To test if the inhibitory effect of HT7 on cocaine locomotion is intensity-dependent, 12 rats were randomly divided into the following three groups; Control, 0.9 m/sec^2^ and 1.3 m/sec^2^ groups. As the intensity (magnitude) of vibration is proportional to the acceleration [22], the intensity of mechanical stimulation by MAI was controlled by adjusting the acceleration of the motors (0.9-1.3 m/sec^2^) by varying the input voltage (1.5 -3 V) (Figure 2C). One min after an injection of cocaine, two different intensities (0.9 or 1.3 m/sec^2^) were applied for 20 sec to the needles inserted at a depth of 3 mm into HT7. Control rats received only cocaine injection without acupuncture treatment. Compared to controls, both intensities significantly attenuated the locomotor response to cocaine (two-way repeated measures ANOVA: group factor F=18.185, p=0.003; time factor F=14.148, p<0.001; interaction F=7.099, p=0.001; Holm–Sidak post-hoc, *p<0.05 vs. Con). There was no significant difference between 0.9 m/sec^2^ and 1.3 m/sec^2^ groups (Figure 2C), suggesting that the inhibitory effect of HT7 might not be proportional to the stimulus intensity or could be triggered supra-threshold. Since a MAI condition of 20 sec in duration and 1.3 m/sec^2^ in intensity produced the best acupuncture effects, the parameter was routinely used in all subsequent experiments, unless stated otherwise. To demonstrate that HT7 inhibition of cocaine locomotor activation was due to stimulation of this acupuncture site specifically, MAI stimulation (20-sec duration, 1.3 m/sec^2^) was applied to LI5, on the opposite side of HT7 on the wrist. MAI LI5 stimulation did not show any significant effect on cocaine locomotion (Figure 2D), indicating that suppression of cocaine locomotion by HT7 MAI stimulation was stimulus point-specific, not generalized motor disturbance at the wrist . 

### Role of Peripheral Nerves in Inhibitory Effects of Acupuncture on Cocaine-induced Locomotion

**Figure 3:**
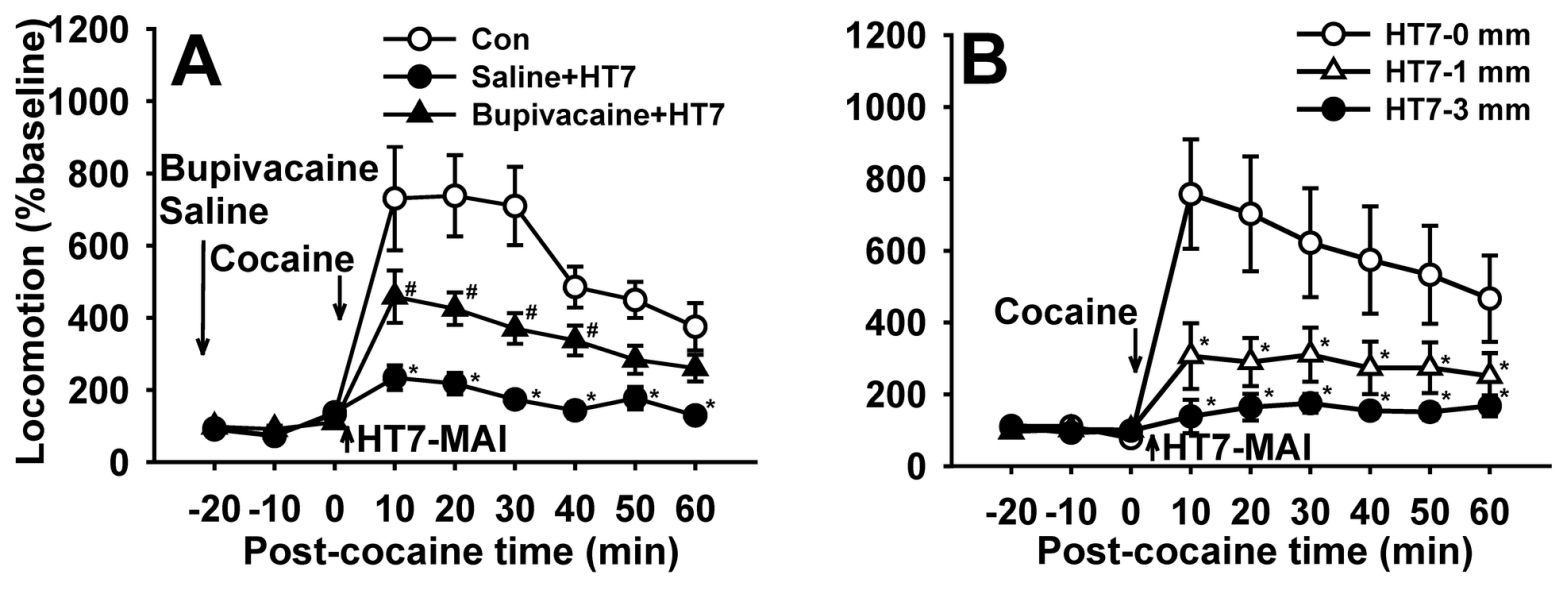
Mediation of peripheral afferents in the inhibitory effects of acupuncture at HT7 on cocaine locomotion. A: Effect of pretreatment with the local anesthetic bupivacaine on the effects of acupuncture at HT7. Inhibition of cocaine locomotion following mechanical stimulation to HT7 was prevented by infiltration around acupoints with bupivacaine. (*P<0.05 vs. Con group (cocaine only); ^#^P<0.05 vs. Saline+HT7 group; Con, n=7; Saline+HT7, n=12; Bupivacaine+HT7, n=12). B: Effect of needling to HT7 at different depths on cocaine locomotion. The HT7 effects are produced by either superficial (1 mm) or deep (3 mm) tissue stimulation (*P<0.05 vs. control; Con, n=11; HT7-1mm, n=12; HT7-3mm, n=12). Data are presented as mean ± SEM and analyzed by two-way repeated ANOVA with Holm-Sidak post hoc comparison.

To identify the role of peripheral nociceptors in the HT7 MAI stimulation, we evaluated the effects of local anesthetic application at acupoints prior to needle stimulation. Rats were randomly assigned to 3 treatment groups: (1) Con, cocaine only; (2) Bupivacaine + HT7. Bupivacaine (30 ul) was injected into HT7 30 min before cocaine and acupuncture; and (3) Saline+HT7, saline instead of bupivacaine was injected. Thirty minutes after bupivacaine injection, the rats were given cocaine (15 mg/kg) followed by a 20-sec mechanical stimulation of the HT7 point. Local injection of bupivacaine at HT7 acupoints tended to inhibit acupuncture effects, compared to saline-treated controls (two-way repeated measures ANOVA: group factor F=18.799, p<0.001; time factor F=29.530, p<0.001; interaction F=7.622, p<0.001; Holm–Sidak post-hoc, *p<0.05 vs. Saline+HT7), suggesting that peripheral nerve afferents mediated the acupuncture effects (Figure 3A). 

To determine the origin of afferent fibers mediating the acupuncture effects, whether they were cutaneous or deep tissue afferents, the acupuncture needle was inserted into HT7 at a depth of either 1 mm or 3 mm and mechanically stimulated. The effects of HT7 on cocaine locomotion were then compared among control (cocaine only), superficial (1mm) and deep (3 mm) acupuncture. There was no difference between the two groups in inhibition of cocaine locomotion (Figure 3B), indicating that the HT7 effect was mediated by both superficial and deep tissue afferents. 

**Figure 4:**
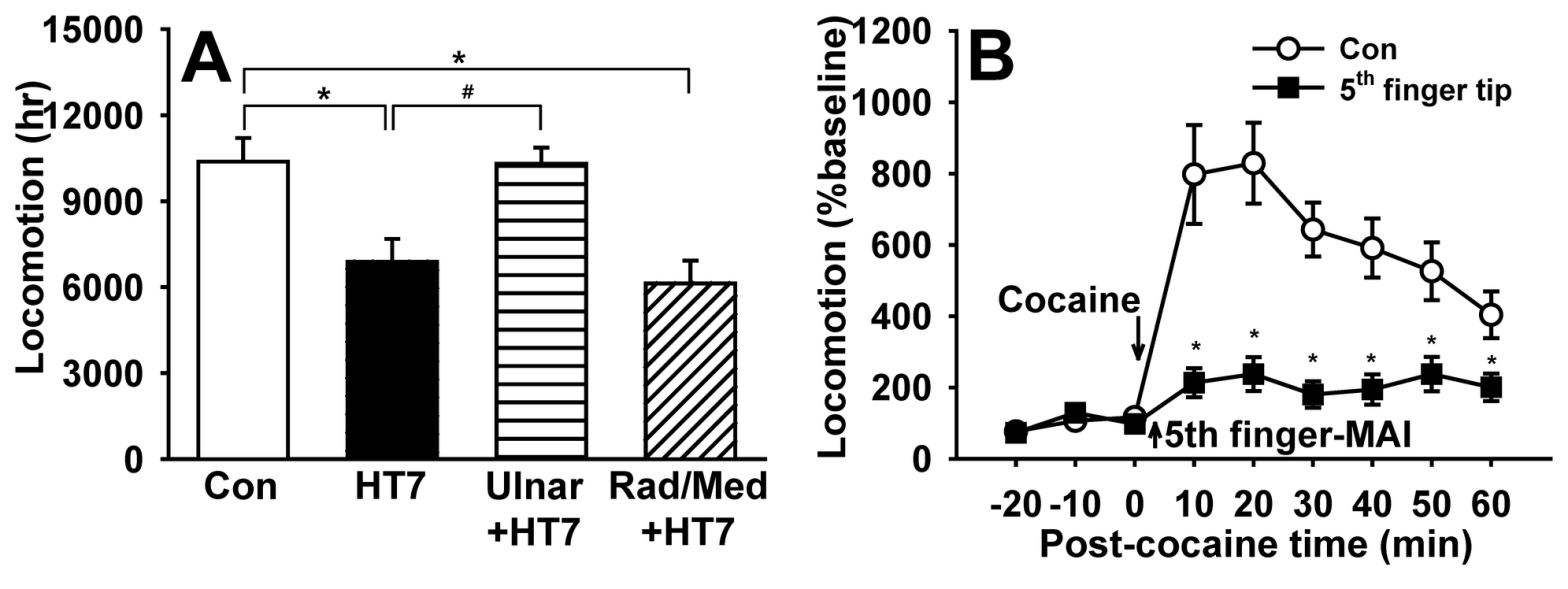
Mediation of the ulnar nerve in the inhibitory effects of HT7 stimulation on cocaine-induced locomotion. A: The inhibitory effects of HT7 on cocaine locomotion are completely blocked by severing the ulnar nerve (Ulnar+HT7), but not by radial and median nerves (Rad/Med+HT7) (Con, n=12; HT7, n=12; Ulnar+HT7, n=11; Rad/Med+HT7, n=9). Data are analyzed by one way ANOVA with Holm-Sidak post hoc comparison. B: Mechanical stimulation of needle inserted into the tip of the 5^th^ digit produced a similar effect). Data are presented as mean ± SEM and analyzed by two-way repeated ANOVA with Holm-Sidak post hoc comparison. * indicates significance at p≤ 0.05.

To determine which peripheral nerve conveyed the acupuncture effects of HT7 stimulation, peripheral nerves innervating the forearm were selectively transected near the elbow 48~72 hours before the acupuncture experiment and the rats were subjected to cocaine treatment followed by acupuncture at HT7. Sectioning the radial and median (Rad/Med+HT7) nerves did not affect the acupuncture effects of HT7, whereas severing the ulnar nerve (Ulnar+HT7) completely blocked MAI HT7 stimulation (Figure 4A), providing compelling evidence that HT7-induced inhibition of cocaine locomotion is mediated by the ulnar nerve. To further confirm mediation by the ulnar nerve, the tip of the 5^th^ digit innervated by the ulnar nerve was mechanically stimulated (20-sec in stimulation time, 1.3 m/sec^2^ in intensity) after needle insertion. Which produced nearly identical effects as HT7 MAI stimulation shown in Figures 1-3 (Figure 4B), indicating the involvement of the ulnar nerve in the inhibitory effects of HT7 stimulation on cocaine-induced locomotion. 

**Figure 5:**
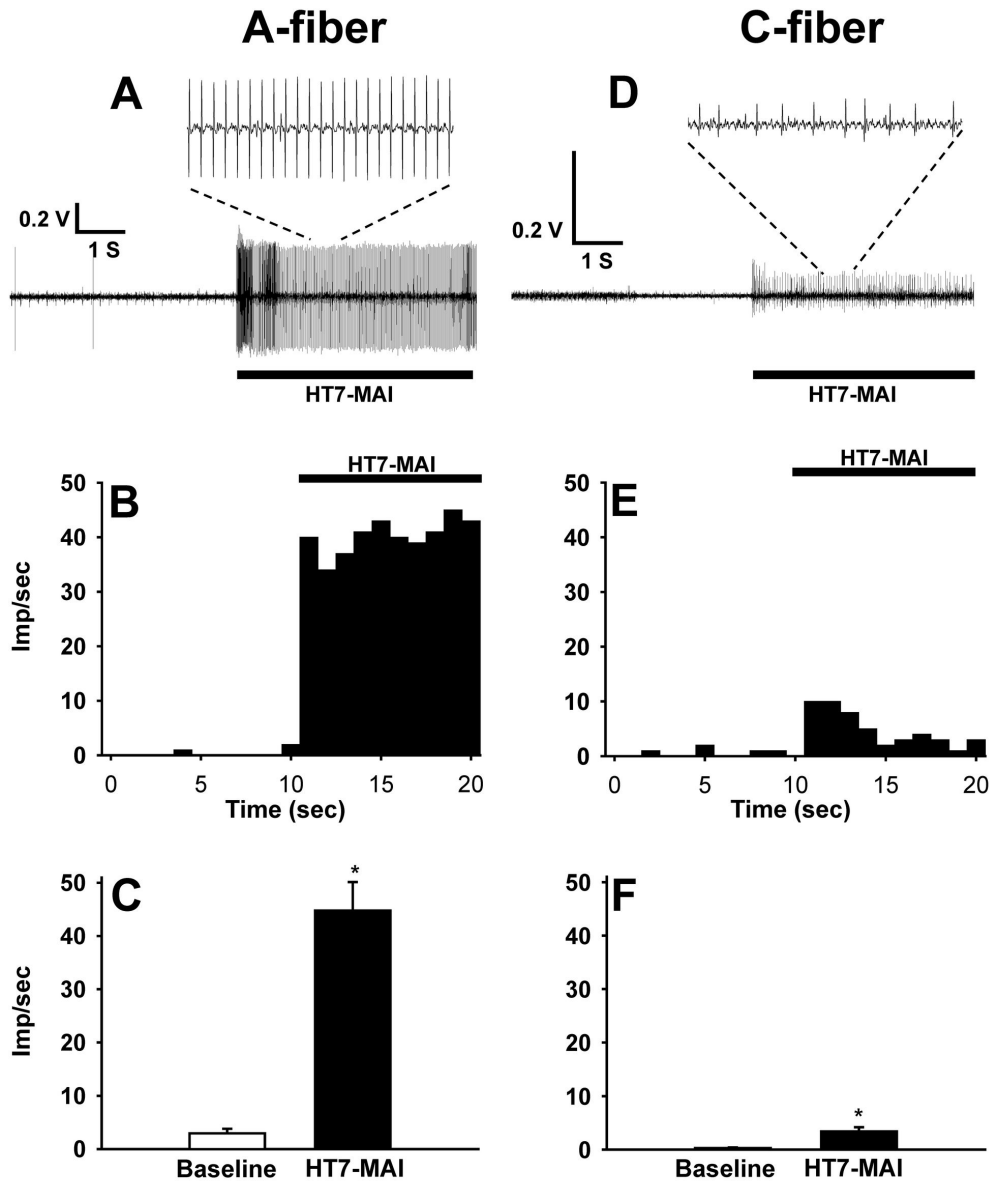
Response of A- or C-fibers during MAI stimulation at HT7. A-C: Somatic afferent responses of Aβ-fibers (n=14) during MAI stimulation of HT7. A, B: A representative single-unit recording (A) and frequency histogram (B) of response to HT7 MAI stimulation (10 sec) in Aβ-fibers. Frequencies of discharge activity dramatically increased during MAI stimulation of HT7, compared to values before MAI stimulation (C). D-F: Somatic afferent responses of C-fibers (n=10) during MAI stimulation of HT7. A, B: A representative single-unit recording (D) and frequency histogram (E) of response to HT7 MAI stimulation (10 sec) in C-fibers. Frequencies of discharge activity significantly increased during MAI stimulation of HT7, compared to values before MAI stimulation (F). Data are presented as mean ± SEM and analyzed by paired Student’s t-test. *p<0.05 compared to baseline (before HT7-MAI).

**Figure 6:**
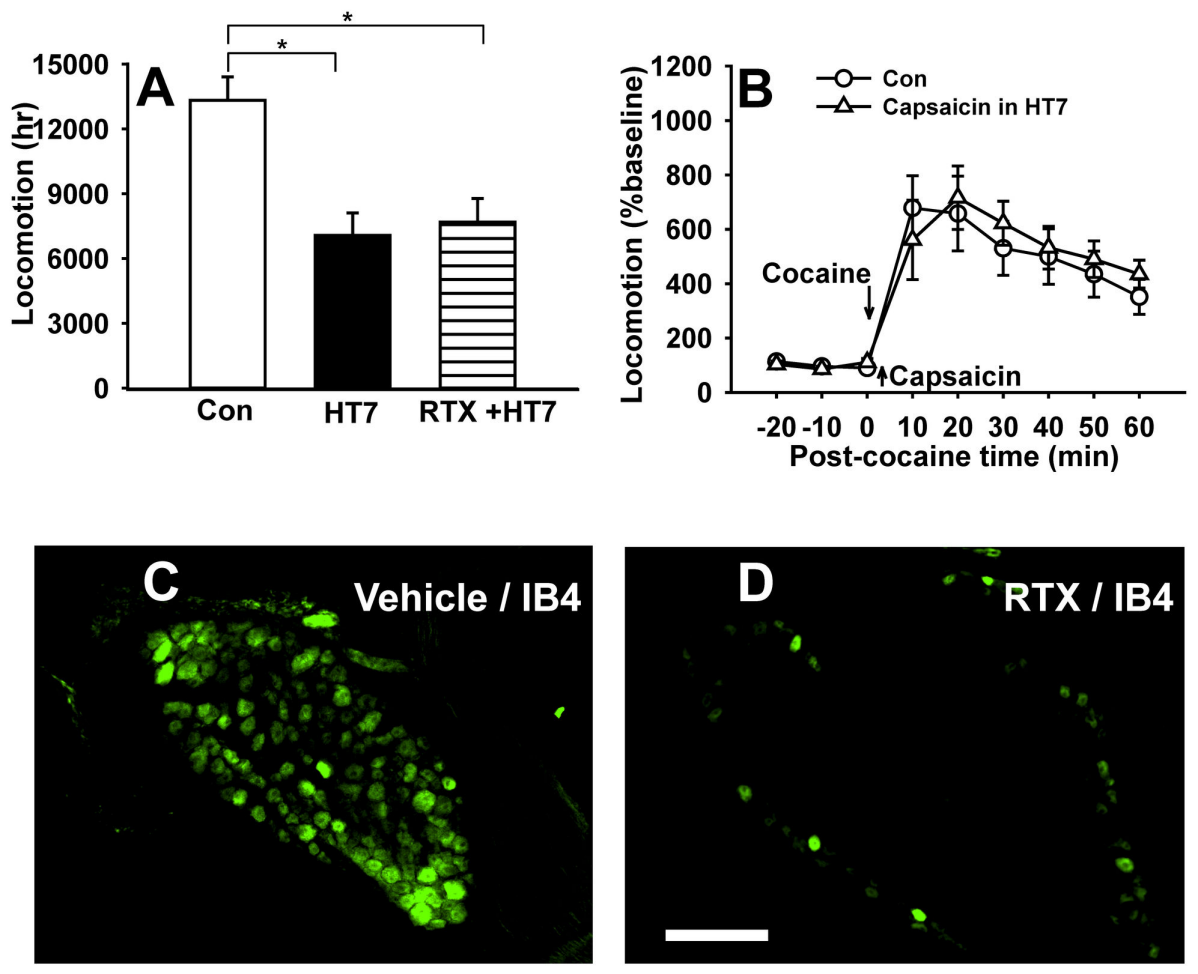
The effects of acupuncture at HT7 require large A-fiber activation of ulnar nerve. A: HT7 effects are not blocked by a perineural injection of RTX (resiniferatoxin; a C/Aδ-fiber inhibitor) into ulnar nerve (RTX+HT7). B: Direct injection of capsaicin into HT7 to stimulate C/Aδ-fibers failed to produce HT7 effects (Capsaicin in HT7). *P<0.05 compared to control (cocaine only). n= 6-8 rats for each group of A-B. Data are presented as mean ± SEM and analyzed by one or two-way repeated ANOVA with Holm-Sidak post hoc comparison. C, D: Epifluorescent images showing IB4-labelled neurons in C8 DRG of a rat injected with IB4 into RTX- and vehicle-treated ulnar nerve. An FITC IB4 tracer (FITC-conjugated isolectin B4) was administered into ulnar nerve on the both side of rats, 48 hours after perineural injection of either RTX or vehicle into right and left ulnar nerve, respectively. Numerous IB4-labelled cells of small size are shown in vehicle-treated DRG (C), whereas a few IB4-labelled neurons are found in the RTX-treated DRG (D). It indicates that retrograde labeling of C-fibers by FITC IB4 tracer is blocked by perineural injection of RTX, but not vehicle. Bar=100 μm.

To determine which fiber groups were activated by HT7 MAI stimulation, single-unit recordings were made from 25 somatic afferents (14 Aβ-, 1 Aδ- and 10 C-fibers) during MAI stimulation of HT7. Although Aβ-fibers responding to mechanical stimulation of HT7 area were recorded easily, we found only 1 Aδ fiber or 10 C-fibers innervating HT7 from 5 rats. One Aδ-fiber recording was excluded from statistical analysis. Compared to basal activity (2.94 ± 0.87 impulses/sec), MAI stimulation at HT7 significantly increased the frequency of Aβ-fiber discharge activity (44.77 ± 5.38 impulses/sec; t=-7.233, df=13, *p<0.001, paired Student’s t-test; Figure 5A-C). The activity of C-fibers during MAI stimulation of HT7 was also enhanced, compared to baseline (3.40 ± 0.80 vs. 0.30 ± 0.10 impulses/sec; t=-3.318, df=9, *p=0.009, paired Student’s t-test; Figure 5 D-F), indicating the activation of both A- and C-fibers by MAI stimulation to HT7. To determine the specific fiber group of the ulnar nerve mediating the acupuncture effects, we tested whether the acupuncture effects on cocaine locomotion were inhibited by pretreatment with resiniferatoxin (RTX), a C/Aδ-fibers blocker. Resiniferatoxin was injected perineurally into the ulnar nerve 24-48 hr before acupuncture treatment. Blocking the ulnar nerve C/Aδ-fibers with RTX did not alter the inhibitory effects of HT7 on cocaine locomotion, suggesting a role for large A-fibers in mediating the effects of HT7 MAI stimulation (Figure 6A). In another set of experiment to confirm RTX blocking of C-fiber, an IB4 tracing study showed that IB4-labeled DRG neurons after RTX treatment were nearly absent (Figure 6D), compared to vehicle-treated controls (Figure 6C), indicating that perineural injection of RTX efficiently blocked nociceptive fibers, especially C-fibers. To further confirm the lack of involvement of C/Aδ-fibers, we evaluated the effects of C/Aδ-fiber activation with capsaicin. After measurement of baseline behavior, capsaicin (0.05 %, 10 ul) was injected at 3 mm deep into the HT7 acupoints using a syringe with a 28-gauge needle to activate nociceptive C/Aδ-fiber afferents from HT7 area and then the locomotion following cocaine injection (Capsaicin in HT7) was compared with control (vehicle+cocaine). Local injection of capsaicin into HT7 did not alter cocaine locomotor activation (Figure 6B), suggesting that the acupuncture effects at HT7 on cocaine locomotion are mediated via large A-fibers in the ulnar nerve. 

**Figure 7:**
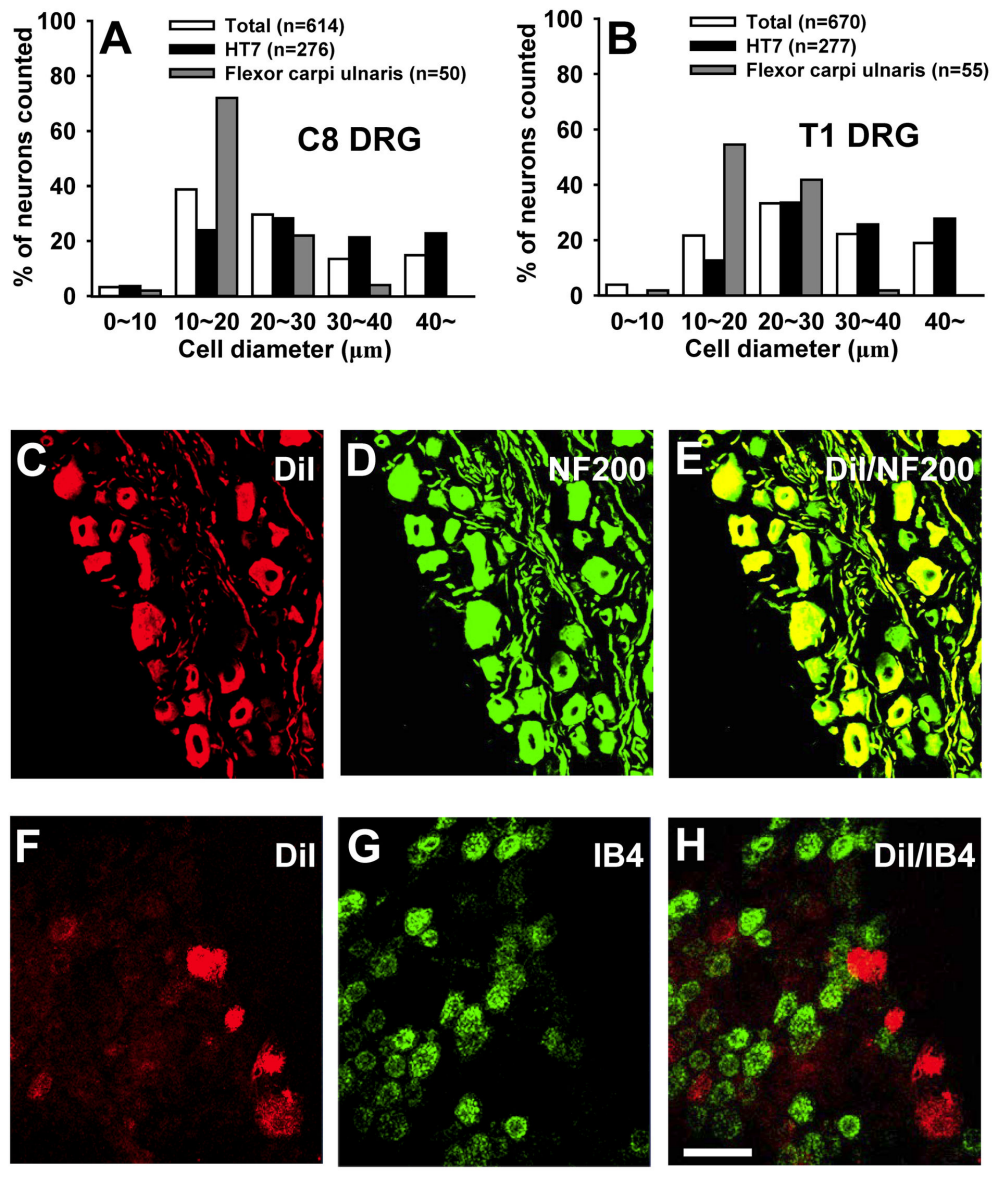
Local afferent fiber composition at HT7 acupoint contains middle- or large-sized A fiber. A, B: Size distribution of C8- and T1-DRG neurons labeled retrogradely after injection of DiI into HT7 acupoints. ‘Total’ indicates histograms of total DRG cell size distribution. And ‘Flexor carpi ulnaris’ indicates histograms the cell size distribution of labeled DRG neurons after injection of DiI into a muscle near HT7. C, D: Most DiI-labeled DRG neurons are double-stained with A-fiber marker NF200 (C), but not C-fiber marker IB4 (D). Bar=100 μm.

**Figure 8:**
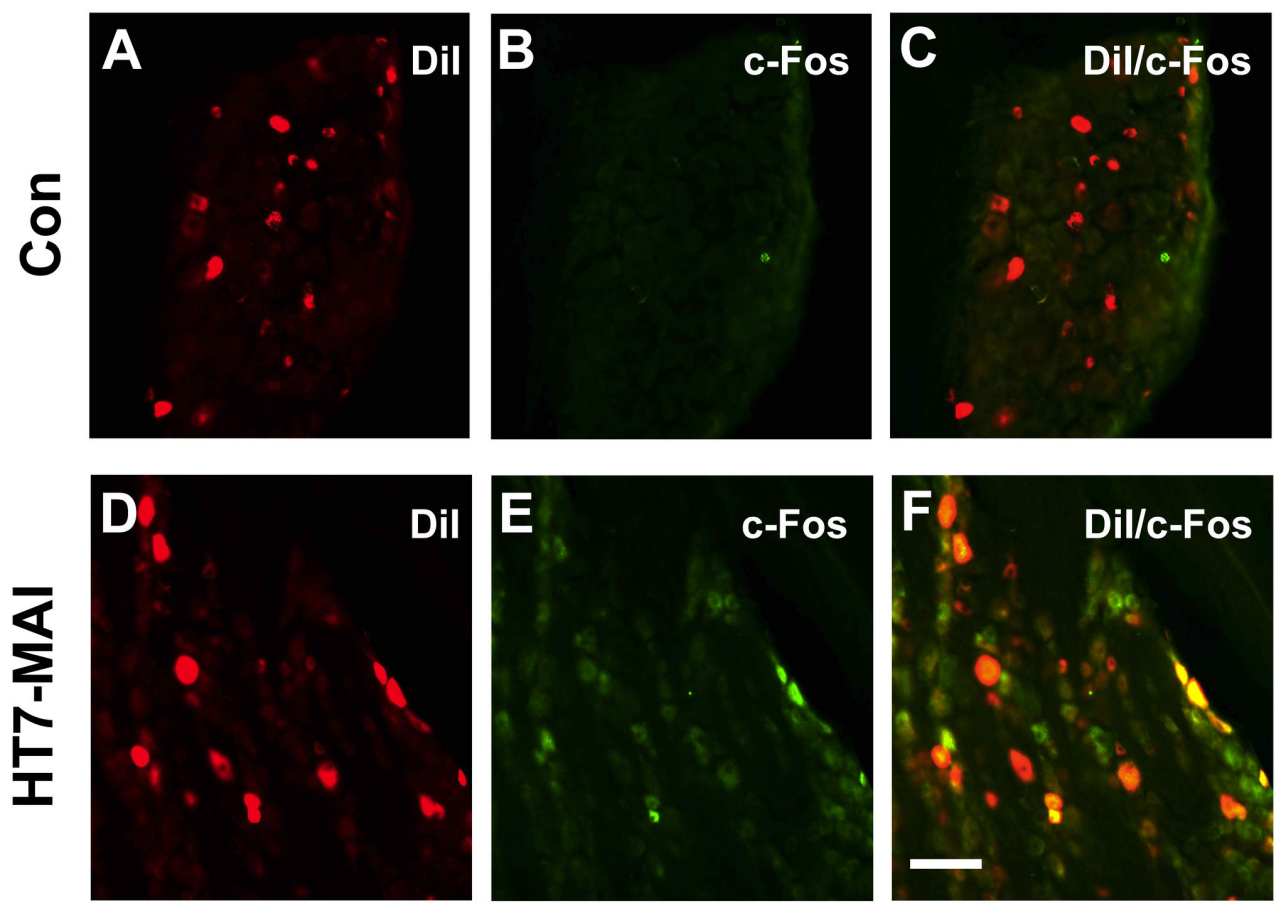
Enhanced c-Fos expression in DiI-labeled DRG neurons following HT7-MAI stimulation. A-C: Immunofluorescent images of the expression of c-Fos (B) in DiI-labeled DRG neurons (A) from control group (no stimulation at HT7). D-F: The expression of c-Fos (E) in DiI-labeled DRG neurons (D) following MAI stimulation at HT7. The numbers of c-Fos positive cells were markedly increased (E) and double labeled cells of c-Fos/DiI were found in the DRG following HT7-MAI stimulation (F), whereas few c-Fos positive cells were seen in untreated DRGs (B). Bar=100 μm.

To identify the types of afferent fibers innervating HT7 area, the retrograde tracer DiI was injected into HT7 acupoints or adjacent muscle (Flexor carpi ulnaris). The size distribution of C8 or T1 DRG neurons labeled by injecting DiI into HT7 showed that DiI-labeled neurons from HT7 are higher in percentage of medium- or large- DRG neurons than those of adjacent muscle and total DRG neurons (Figure 7A,B). Most DRG neurons labeled from DiI injection into HT7 also showed positive immunoreactivity for NF200, a marker for large myelinated A-fiber neurons (Figure 7C-E), but not IB4, a C-fiber marker (Figure 7F-H), demonstrating that HT7 acupoints are innervated by relatively large myelinated A-fibers of the ulnar nerve. To identify if these DiI-labeled neurons were responsive to HT7 MAI stimulation, only the left HT7 was stimulated with MAI (60 sec) while the right HT7 remained intact without stimulation. The expression of c-Fos, a marker of neuronal activation, in DiI-labeled neurons was compared between left and right DRGs. As shown in Figure 8, only a few c-Fos-labeled neurons were found in DiI-labeled DRGs from untreated right side (n=4, Con, 13.97 ± 0.06 %), whereas c-Fos expression in DiI-labeled DRGs (left side) was significantly increased by HT7 MAI stimulation (n=4, HT7-MAI, 56.20 ± 0.28 %, p<0.05 vs. Con), indicating that DiI-labeled DRG neurons are activated by HT7 MAI stimulation. 

**Figure 9:**
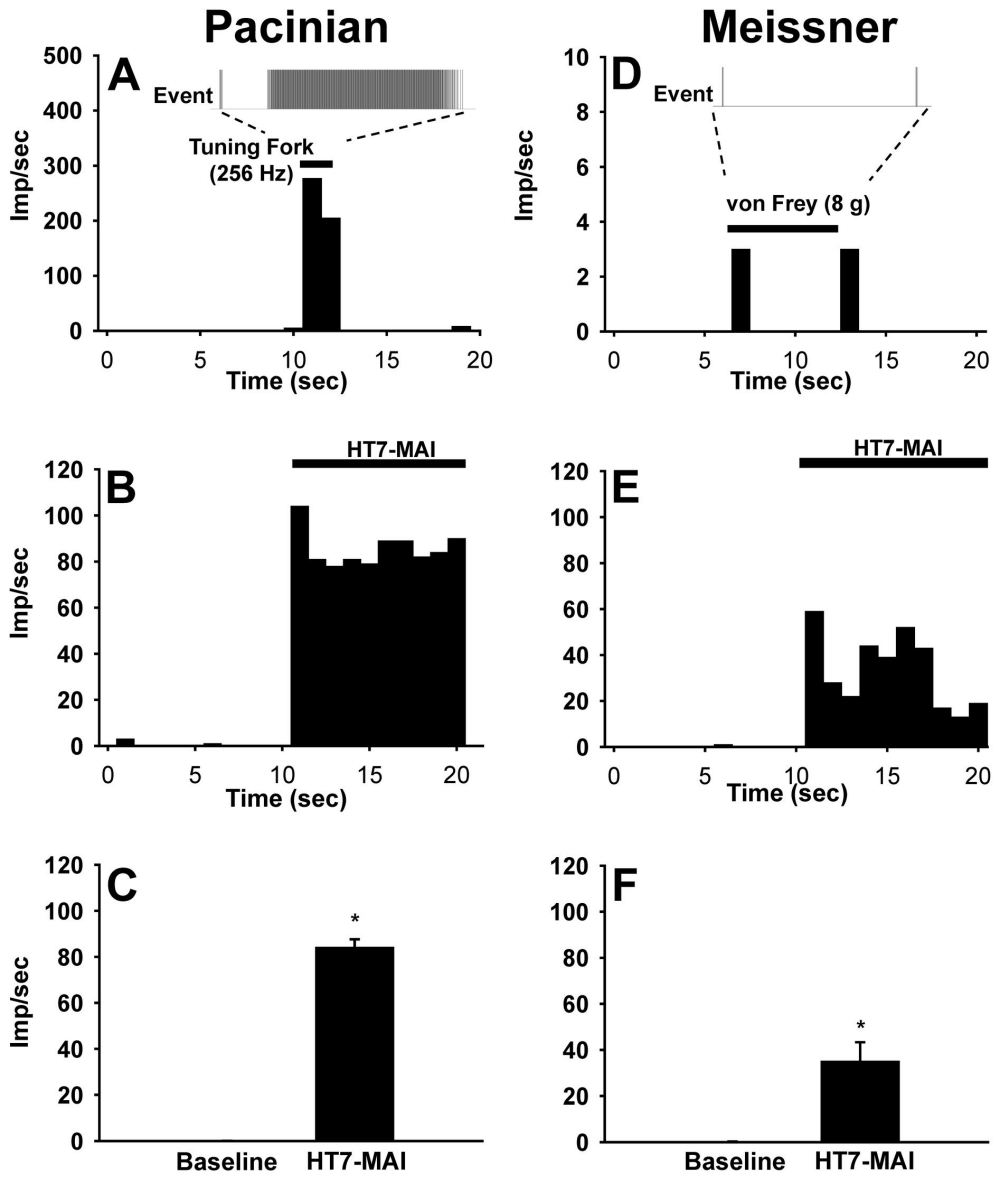
Activation of Pacinian and Meissner corpuscle fibers during HT7-MAI stimulation. A-C: Somatic afferent responses of Pacinian corpuscle fibers (n=9) during MAI stimulation of HT7. A: An example of event traces and frequency histogram of Pacinian corpuscle fiber during high frequencies stimuli with tuning fork (256 Hz). B: Frequency histogram of response to HT7 MAI stimulation (10 sec). Frequencies of discharge activity dramatically increased during MAI stimulation of HT7, compared to values before MAI stimulation (C). D-F: Somatic afferent responses of Meissner corpuscle fibers (n=8) during MAI stimulation of HT7. D: An example of event traces and frequency histogram of Meissner corpuscle fiber during the sustained pressure on HT7 for about 7 sec with von Frey 8 gram force. E: Frequency histogram of response to HT7 MAI stimulation (10 sec). Frequencies of discharge activity significantly increased during MAI stimulation of HT7, compared to values before MAI stimulation (F). Data are presented as mean ± SEM and analyzed by paired Student’s t-test. * indicates significance at p≤ 0.05.

**Figure 10:**
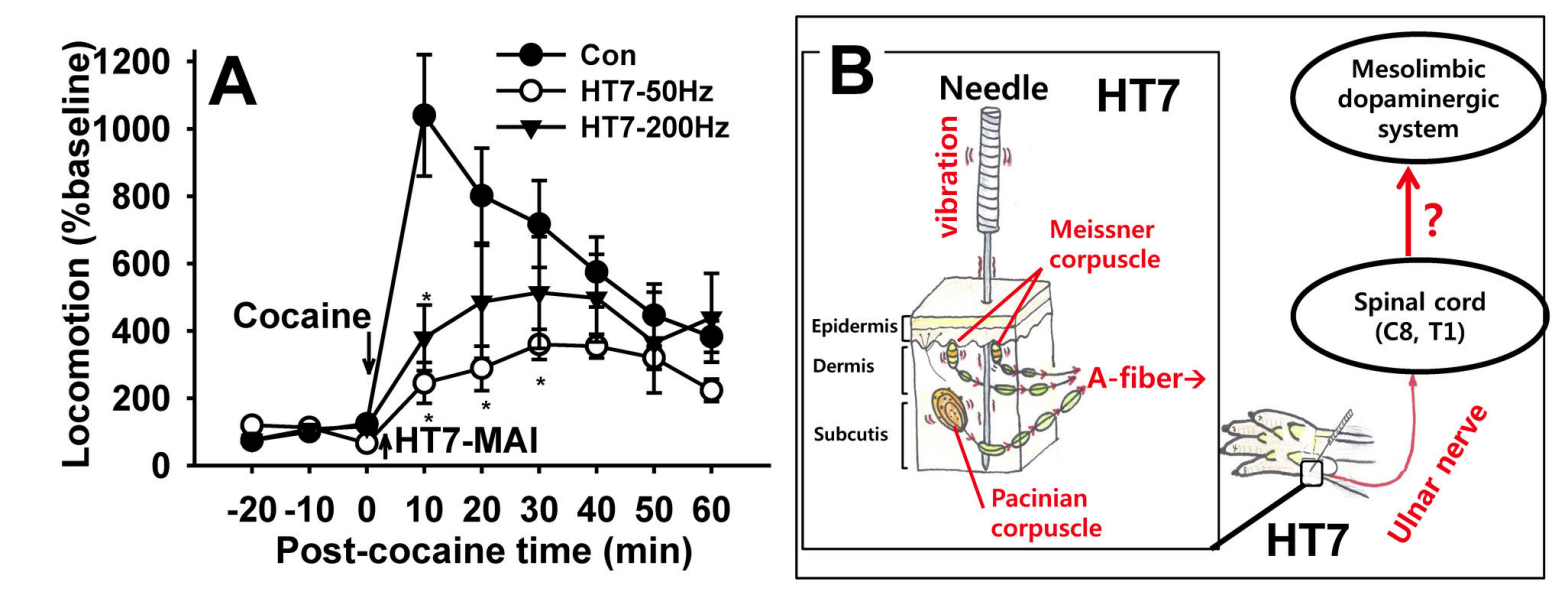
Possible involvement of mechanoreceptors, Pacinian and Meissner corpuscles, in HT7 effects. A: The HT7 effects are generated by mechanical stimulation applied to HT7 at either high (200 Hz) or low (50 Hz) frequencies. *p<0.05 compared to control (cocaine only). n= 6-8 rats for each group. B: A diagrammatic representation of the proposed hypothesis that the effects of manual acupuncture at HT7 on cocaine-induced locomotion are mediated via A-fiber activation of ulnar nerve originating from superficial and deep tissue. Data are presented as mean ± SEM and analyzed by two-way repeated ANOVA with Holm-Sidak post hoc comparison.

Finally, to investigate which types of peripheral receptor of A-fiber, Pacinian or Meissner corpuscles, initiate the acupuncture effects, it was tested whether Pacinian or Meissener corpuscles were activated by MAI stimulation (85 Hz, 1.3 m/sec^2^) at HT7 or whether mechanical stimulation to HT7 at high (200 Hz) or low (50 Hz) frequency reduced cocaine locomotion. Pacinian fibers (n=9) that responded to high frequency stimulation with a tuning fork (256 Hz) were isolated as described previously [18] (Figure 9A). Fibers from Pacinian corpuscles that responded to HT7 MAI evinced initial spiking followed by subsequent regular discharges to 83.85 ± 3.78 imp/sec from basal activity of 0.07 ± 0.03 imp/sec t=-20.884, df=8, *p<0.001, paired Student’s t-test; Figure 9B,C). On the other hand, fibers from Meissner corpuscles were characterized by transient responses at the beginning and end of the sustained pressure with von Frey filament (8 gram force) on the HT7 acupoint (Figure 9D). In Meissner corpuscle fibers (n=8), HT7 MAI stimulation evoked spiking to 34.73 ± 8.62 imp/sec from baseline (0.17 ± 0.08 imp/sec; t=-3.773, df=7, *p=0.007, paired Student’s t-test; Figure 9E,F), suggesting that large A-fiber receptors, Pacinian and Meissner corpuscles, were activated by HT7 MAI. To further determine whether activation of Pacinian and Meissener corpuscles mediates the HT7 effects, the effects of 200 or 50 Hz frequencies stimulation of HT7 on cocaine locomotion were examined. As shown in Figure 10A, HT7 stimulation at either 50 or 200 Hz inhibited cocaine locomotion, indicating the involvement of both Pacinian and Meissner corpuscles in HT7 acupuncture. 

## Discussion

Mechanical stimulation of HT7 suppressed cocaine-induced locomotion in a time-dependent manner. The HT7 effects were produced by either superficial or deep stimulation to HT7 and also inhibited by local anesthetic around HT7 points. The HT7 effect was blocked by severing the ulnar nerve and mimicked by stimulation of 5^th^ digit innervated by the ulnar nerve. Furthermore, it was not affected by either blockade of C/Aδ-fiber in the ulnar nerve or direct activation of C/Aδ-fibers lying adjacent to HT7 points. DiI retrograde tracing revealed a high density of large myelinated A-fibers in HT7 area. HT7 stimulation at either 50 or 200 Hz was effective in suppressing cocaine locomotion. These results suggest that mechanical (manual) stimulation associated with HT7 acupuncture activates both Pacinian and Meissner corpuscles and the signals of acupuncture are conveyed via large A-fibers in the ulnar nerve, which produce the inhibitory effects on cocaine locomotion. 

### 1. Acupuncture Device Mimicking Human Mechanical Stimulation

The use of acupuncture in oriental medicine traces back to several thousand years and has been used successfully to treat various conditions. Historically, acupuncture needles are manually stimulated by the hand of the acupuncturist. Over the past several decades, manual acupuncture (MA) has been substituted with electroacupuncture (EA), since EA has the advantage of better control of stimulation frequency and intensity, resulting in high reproducibility and lower individual variations among physicians [21]. While a number of studies have suggested that both MA and EA may recruit identical peripheral or central mechanisms [11,23], several studies have implicated that the effectiveness or the underlying mechanisms between MA and EA are different. For example, in functional brain mapping study, EA produces more widespread signal increase than MA [21]. EA and MA differentially affect salivary flow through releases of different types of neuropeptides in healthy subjects [24]. EA releases both β-endorphin and adrenocorticotropic hormone in the blood, whereas MA releases β-endorphin only [25]. Although EA was developed to solve some of the control issues of MA, and is popular in acupuncture research, EA’s electrical effects may be different from the basic mechanical mechanism of conventional acupuncture, which has long been used in Asian countries. To investigate the basic mechanisms of MA, we used a mechanical acupuncture instrument (MAI) which was able to apply quantitative mechanical stimulation. As proof of concept, we explored the peripheral mechanisms underlying acupuncture’s effectiveness in an animal model of acute cocaine. The increase of locomotion following acute cocaine was inhibited in a time-dependent manner reaching maximum effect at 20 sec duration. The stimulus-time (20 sec) for maximal effects of MAI was significantly shorter than typical stimulus-time for EA (about 30 min [11,26]). Our laboratory has previously demonstrated that manual stimulation for 60-sec at HT7 has potent inhibitory effects in various addiction models [3,5,7]. Indeed, short stimulus-time MA has also been demonstrated to be effective in previous experiments [27-29]. Accordingly, it is reasonable to propose that rapidly-adapting peripheral systems may be recruited during MA under certain conditions. Our findings suggest that rapidly-adapting mechanoreceptors, such as Pacinian and Meissner corpuscles, are the likely candidates for mediating MA (Figure 6A). 

### 2. HT7 Acupuncture Involves Both Superficial and Deep Nerve Activation

Acupuncture effects may be mediated through neuronal or traditional Qi energies [13,26,30]. In the present study, the acupuncture-mediated inhibition of cocaine-induced locomotion was blocked by local anesthesia at acupoints with bupivacaine, suggesting that the afferent signals associated with acupuncture are conveyed via nerve fibers. This is consistent with previous studies showing that acupuncture effects are blocked by pretreatment with local anesthetic drugs around the acupoints [31,32]. The transmission of acupuncture afferent signals through peripheral nerve is further supported by our findings that HT7 MAI stimulation was ineffective in inhibiting cocaine-induced locomotion after severing the ulnar nerve. Questions have been raised regarding the origin of afferent signals with acupuncture: Which are superficial or deep tissue afferents? In previous studies, EA analgesia in a rat ankle-sprain model is produced by deep needling (3 mm) at SI5, but not shallow (0.5 mm), indicating the generation of acupuncture afferent signals from deep tissues [33]. On the other hand, Sato and his colleagues reported that the acupuncture inhibition of micturition contractions of the urinary bladder are produced by acupuncture-like stimulation of either superficial or deep tissues [34]. Our present data showed that acupuncture inhibition of cocaine locomotion was elicited by either superficial (1 mm) or deep (3 mm) acupuncture at HT7.

### 3. HT7 Inhibition of Cocaine-induced locomotion Requires Activation of A-fibers in the ulnar nerve

Acupuncture suppression of cocaine-induced locomotor activity was completely abolished after severing the ulnar nerve and mimicked by mechanical stimulation inserted into the tip of the 5^th^ digit, which is innervated by ulnar nerve [35]. This is consistent with previous findings that the responses elicited by stimulation of acupoints are blocked when the nerve lying under the acupoint is cut [33,36], and direct stimulation of nerve innervating acupoints also produces a similar effect as that of acupuncture [33,37]. It suggests that the nerve innervating the acupoint plays an important role in producing the acupuncture effects. Our present data that stimulation of 5^th^ digit caused a similar response to HT7 MAI stimulation may also suggest that the nerve, not acupoints along the nerve, is important in producing specific responses. It is still controversial what types of afferent nerve fibers are activated by MA or EA. For example, Guowei et al. suggest that the afferent signals from acupuncture at ST36 for producing analgesia are conveyed through A-fibers of the perineal nerve [10]. Our previous study reported that Aδ fibers mediate analgesic effects of EA at SI5 in ankle sprain model [33]. It has been shown that MA and EA applied to PC5-6 acupoints activate Aδ and C fibers to evoke cardiovascular effects [11] and blockade of C-fiber afferents using neonatal capsaicin completely eliminates the cardiovascular effects of EA [38], suggesting critical role of Aδ or C fibers in acupuncture-mediated cardiovascular regulatory effects. In the present study, perineural injection of a C/Aδ-fiber blocker resiniferatoxin into ulnar nerve did not alter HT7 MAI acupuncture. In turn, direct injection of C/Aδ-fiber activator capsaicin into HT7 did not elicit HT7-like effects. Moreover, our retrograde study to determine fiber types innervating HT7 showed that HT7 is located in the area innervated by relatively large myelinated A-fiber, suggesting that HT7 area has a local condition sensitive to mechanical stimuli such as vibration, touch, pressure and stretch. Our present findings suggest the large A-fibers of ulnar nerve play a pivotal role in producing the inhibitory effects of manual acupuncture at HT7 on cocaine locomotion. Taken together, the types of nerve fibers activated during acupuncture stimulation may depend on animal models, stimulation paradigms and acupoints used. 

### 4. HT7 Inhibition of Cocaine-induced locomotion Involves Activation of Pacinian and Meissner Corpuscles

Which receptors of large A-fibers might initiate afferent signals during MA to produce the inhibitory effects on cocaine locomotion? Vibration stimuli are transmitted by two main types of mechanoreceptors, Pacinian and Meissner corpuscles that differ in physical properties and anatomical location. Pacinian corpuscles are most sensitive to high frequencies of 200 Hz, whereas Meissner corpuscles are responsive to lower frequencies around 10~50 Hz. Pacinian corpuscles are found in the deep subcutaneous tissues, the tendons of the muscles and the ligaments of joints, while Meissner corpuscles are located in the superficial portion of the dermis [39,40]. In the present study, mechanical stimulation applied to HT7 at frequencies of either 50 or 200 Hz produced acupuncture effects, although those of 50 Hz had stronger effects, suggesting the involvement of both Pacinian and Meissner corpuscles in skin and deep tissues in initiating the acupuncture effects after mechanical stimulation of HT7. Furthermore, our findings in Figure 3B showed that acupuncture effects were produced by either superficial (1 mm) or deep (3 mm) stimulation. We assumed that the effects by superficial or deep acupuncture may be due to activation of Meissner and Pacinian corpuscles, respectively. We have previously shown that MA at HT7 modulates GABAergic neurons in VTA and dopaminergic transmission in the NAc [2,3]. In conclusion, brief, manual acupuncture at HT7 suppresses cocaine-induced locomotor activity in a stimulus, time-dependent manner. The present data suggest that the superficial or deep afferents of ulnar nerve are activated during mechanical stimulation of HT7 and the signals are transmitted via large A-fiber of ulnar nerve to produce the inhibitory effects on cocaine locomotion (Figure 10B). 
